# Digital Teaching and Digital Medicine: A national initiative is needed

**DOI:** 10.3205/zma001189

**Published:** 2018-08-15

**Authors:** Martin Haag, Christoph Igel, Martin R. Fischer, German Medical Education Society (GMA), Committee “Digitization – Technology-Assisted Learning and Teaching”, Joint working group “Technology-enhanced Teaching and Learning in Medicine (TeLL)” of the German Association for Medical Informatics, Biometry and Epidemiology (gmds) and the German Informatics Society (GI)

**Affiliations:** 1 Heilbronn University, GECKO Institute for Medicine, Informatics and Economics, Heilbronn, Germany; 2 German Research Center for Artificial Intelligence, Educational Technology Lab, Berlin, Germany; 3 University Hospital, LMU Munich, Institute for Medical Education, Munich, Germany

**Keywords:** Digital Teaching, Digital Transformation, Digital Medicine, Open Educational Resources

## Abstract

The advancing digitization of the healthcare system requires that in the future digital skills should be communicated to students much more. In addition, digital teaching and learning technologies should be used wherever they offer real benefits over other training scenarios. To meet these challenges, it needs a national initiative “Medical Education in the Digital Age”, which should be led by the Society for Medical Education and the German Association for Medical Informatics, Biometry and Epidemiology.

## Zusammenfassung

Die voranschreitende Digitalisierung des Gesundheitssystems macht es erforderlich, in Zukunft digitale Kompetenzen deutlich stärker an die Studierenden zu vermitteln. Darüber hinaus sollten digitale Lehr- und Lerntechnologien überall eingesetzt werden, wo diese einen echten Nutzen gegenüber anderen Ausbildungsszenarien bieten. Um diese Herausforderungen zu meistern, benötigt es einer nationalen Initiative „Medizinausbildung im digitalen Zeitalter“, die von der Gesellschaft für Medizinische Ausbildung und der Deutschen Gesellschaft für Medizinische Informatik, Biometrie und Epidemiologie angeführt werden sollte.

## Introduction

While the digital transformation of the health care system is progressing, the required knowledge in digital medicine in most universities seems to be insufficiently communicated and existing digital tools are not adequately used.

### What are the challenges?

1. *Communicate Digital Competencies:* Medical students are not adequately prepared to master existing and future challenges of digital medicine. This includes, in particular, the selection and deliberate use of digital tools and systems and their critical evaluation, with regard to questions of data protection and ethics for the benefit of the patients [1]. In this process, systems driven by artificial intelligence become themselves diagnosing agents in the system. The role of patients as the owner and guardian of their health data will fundamentally change and transform the professional roles in health care – how radical cannot yet be estimated.
2. *Making meaningful use of digital teaching and learning technologies:* Although in many contexts learning is effectively supported by the use of digital teaching and learning technologies [2], the use of modern digital teaching/learning and testing technologies is mostly only selective.

Unfortunately, a nationwide and rapid adaptation of university teaching to the current challenges cannot be identified. There are multiple reasons for that. In addition to insufficient or missing infrastructure and lack of training opportunities for teachers, the high additional burden on teachers through patient care and research should be mentioned. In addition, there are unfavorable legal framework conditions that impede innovation.

While the subject of digitization of universities or higher education is already well addressed across disciplines through initiatives such as the Higher Education Forum Digitization (“Hochschulforum Digitalisierung”) or the Intelligent Education Networks Expert Group at the National IT Summit, no national initiative currently addresses the specific requirements of medical education and the healthcare system. So far, only individual initiatives of lecturers exist, such as the project “Medicine in the Digital Age” funded by the Stifterverband and the Carl Zeiss Foundation [1], in which a “Curriculum 4.0” is being developed.

## Medical education in the digital age

It takes a concerted effort to overcome existing barriers and to qualify medical students and medical teachers for the medicine of the future. In addition to the medical faculties and specialist societies, federal and state governments are required to provide a legal framework that does not inhibit the creation and use of digital teaching objects but promotes them.

What is needed is a digital infrastructure on the Internet that enables educators to collaboratively develop, use and develop content. In addition, it must be quickly and easily possible to identify appropriate content for one's own teaching, for example, by tagging content based on keyword catalogs such as the NKLM [http://www.nklm.de]. In the creation of such an infrastructure, the intensive participation of teachers, (medical) informatics and education researchers is essential. In this context, the BMBF's previous project funding in the field of shared content and shared services is heading in the right direction, but must be continued and significantly strengthened in order to help open educational resources (OER) achieve a breakthrough in the broad field of medical education [3]. In this context, copyright adjustments are also required and a comprehensive open access and open content strategy must be created. IT solutions that can be integrated into local infrastructures without high costs are absolutely essential.

Encouraging collaboration on the labor-intensive and cost-intensive development of digital content across academic boundaries requires an incentive and rewards system for educators and institutions. The reliable recognition of digital teaching performance is helpful here, as shown in this issue [4]. The provision of self-created content must also be of real benefit to the lecturers themselves and must not be associated with the concern, possibly to violate the copyright. Here, suitable basic conditions must be created by the universities, federal states and the federal government.

To support the teachers, significantly more counseling and support services must be provided. For cost and efficiency reasons, a mix of advisory and support measures should be used locally and centrally on the Internet.

The Medical Informatics Initiative [http://www.medizininformatik-initiative.de] tries to find a way with big effort so that research results can find their way more quickly into the treatment of patients. For example, new insights are to be gained by analyzing large amounts of data. A mutual integration of current projects with research, education and health care can be of great benefit here [5]. Educational Data Mining and Learning Analytics also deal with the use of existing (large) amounts of data. Existing or still to be collected data can be used to give teachers better insights into their learning groups and to be able to better adapt teaching to the actual needs. Clear legal framework conditions and recommendations for action for data protection officers and media or service centers at universities are needed here [3].

The Master Plan for Medical Studies 2020 does not address the topic of digital transformation [1], it needs to be improved. Although the National Competency-Based Learning Catalog (NKLM) contains a number of relevant learning objectives in the field of Medical Informatics, these must be supplemented to meet the requirements of future-oriented education.

## Conclusions

The digitization of the health care system is progressing inexorably, and this fact must be taken more into account in both teaching content and teaching. A national initiative “Medical Education in the Digital Age” is needed to bring about the urgently needed changes. Those responsible in faculties and ministries must take greater account of digitization and initiate appropriate measures. The German Association for Medical Education (GMA) as well as the German Association for Medical Informatics, Biometry and Epidemiology (gmds) should promote the necessary discussions and jointly lead a national initiative “Medical Education in the Digital Age”.

## Competing interests

The authors declare that they have no competing interests. 
